# Prognostic relevance of SMC family gene expression in human sarcoma

**DOI:** 10.18632/aging.202455

**Published:** 2020-12-30

**Authors:** Jian Zhou, Gen Wu, Zhongyi Tong, Jingjing Sun, Jing Su, Ziqin Cao, Yingquan Luo, Wanchun Wang

**Affiliations:** 1 Department of Orthopedics, The Second Xiangya Hospital, Central South University, Changsha 410011, Hunan, China; 2 Clinical Medicine Eight-year Program, 02 Class, 2014 Grade, Central South University, Changsha 410013, Hunan Province, China; 3 Department of Pathology, The Second Xiangya Hospital, Central South University, Changsha 410011, Hunan, China; 4 Department of Anesthesiology, Second Affiliated Hospital, School of Medicine, Zhejiang University, Hangzhou 310009, Zhejiang, China; 5 The Center for Medical Genetics, School of Life Science, Central South University, Changsha 410008, China; 6 Department of General Medicine, The Second Xiangya Hospital, Central South University, Changsha 410011, Hunan, China

**Keywords:** sarcoma, bioinformatics analysis, prognosis, SMC, expression

## Abstract

Objective: To explore the prognostic value of the expression of genes encoding structural maintenance of chromosomes (SMCs) in human sarcoma.

Results: We found that the levels of SMC1A, SMC2, SMC3, SMC4, SMC5 and SMC6 mRNA were all higher in most tumors compared to normal tissues, and especially in sarcoma. According to the Cancer Cell Line Encyclopedia (CCLE), SMC1A, SMC2, SMC3, SMC4, SMC5 and SMC6 are also highly expressed in sarcoma cell lines. Results of Gene Expression Profiling Interactive Analysis (GEPIA) indicated that high expression of SMC1A was significantly related to poor overall survival (OS) (p<0.05) and disease-free survival (DFS) in sarcoma (p<0.05). Additionally, strong expression of SMC2 was significantly related to poor OS in sarcoma (p<0.05). In contrast, SMC3, SMC4, SMC5, and SMC6 expression had no significant impact on OS or DFS in sarcoma.

Conclusions: Expression of SMC family members is significantly different in sarcoma relative to normal tissues, and SMC1A and SMC2 may be useful as prognostic biomarkers.

Methods: We performed a detailed comparison of cancer and normal tissues regarding the expression levels of mRNA for SMC family members in various cancers including sarcoma through ONCOMINE and GEPIA (Gene Expression Profile Interactive Analysis) databases.

## INTRODUCTION

Sarcomas constitute a group of malignant tumors that can differentiate into many different tissue lineages including fat, bone, fiber and muscle. In the United States, sarcomas account for 1% of newly diagnosed tumors and tumor-related deaths. These tumors were historically categorized as bone or soft tissue sarcomas, but more recent molecular classification divides them into genetically complex or genetically simple [1]. Because of their molecular and histological heterogeneity, sarcomas are difficult to diagnose. Therefore, new biomarkers are needed to provide individualized treatment and improve prognosis.

SMC proteins are encoded by the SMC family genes, and are required for the proper condensation and segregation of mitotic chromosomes. SMC proteins have been shown to be involved in DNA repair, genetic recombination, sex-chromosome dosage compensation, sister chromatid cohesion and chromosome condensation [2]. The SMC proteins take the hinge region as their axis and rely on the interaction of the two helical domains to fold in an antiparallel manner to form a rod-shaped molecule with a hinge region at one end and a head region binding ATP at the other. The hinge regions of two SMC proteins interact to mediate dimerization and take on a V-shaped molecular conformation. This family includes 6 proteins designated SMC1-6, of which the SMC1-SMC3 dimer forms the core of the cohesion protein complex and mediates the adhesion of sister chromatids. SMC2-SMC4 is the core component of the condensin protein complex, which is involved in the assembly and separation of chromosomes. The start sequence of SMC5-SMC6 is slightly different from SMC1-SMC3, forming a third complex, which mainly plays a role in DNA replication and checkpoint response [3].

SMCs are believed to have distinct and complex roles in different tumors. Abnormal expression of the SMC1A and SMC3 genes, which encode important components of the cohesion protein complex, is found in a variety of human malignancies. For example, it was reported that phosphorylated SMC1A was overexpressed in hepatocellular carcinoma cells, and that this was significantly associated with poor prognosis [4]. SMC3 was also reported to be overexpressed in colorectal cancer tissues and A549 lung cancer cells [5]. SMC2 is an important part of the condensate complex, which plays a significant role in chromatin packaging before cell division and in handling DNA damage; it is required for correct chromosome separation and maintenance of chromosome stability. SMC2 plays a dual role in the development of cancer. For example, SMC2 is involved in mitotic cell division and new evidence indicates that it might have a cancer-promoting effect. Previous reports showed that knocking down the SMC2 gene could inhibit tumor growth in colorectal cancer and increase apoptosis of neuroblastoma cells. Studies have also shown that the level of expression of SMC2 mRNA in human pancreatic cancer tissue is clearly higher than in the corresponding non-tumor tissue [6]. SMC4 is a chromosomal ATPase, with a highly conserved nucleotide-binding domain at both its N- and C-terminals. The main function of SMC4 is assisting in the process of chromosomal transition from loose interphase chromatin to the agglomerated state, and in sister chromatid separation during division. In addition, SMC4 also plays a significant role in the non-dividing phase of the cell cycle, including the maintenance of gene repression, heterochromatin organization and DNA repair. Recent studies have shown that the expression level of SMC4 is abnormally high in liver cancer, breast cancer [7], and colon cancer. Moreover, SMC4 can promote the growth of liver cancer and colon cancer. The risk of lung adenocarcinoma-related death in patients with high SMC4 expression was about 1.5-fold that of those with lower expression [8]. Strong expression of SMC4 was also significantly related to the poor OS of patients with lung adenocarcinoma, indicating that it might be an independent poor prognostic factor for this tumor. The SMC5/6 complex plays an important role in the DNA damage repair pathway. SMC5/6 is rapidly recruited to DSB and in turn recruits other SMC members. Previous studies showed SMC5/6 also assists in the maintenance of telomere length in ALT cancer cells, whereby these cells acquire unlimited replicative potential [9].

Several studies have confirmed that the level of expression of SMC family member genes in tumor cells is dysregulated and related to the clinicopathological characteristics of the tumor. To the best of our knowledge, bioinformatics analyses to shed light on the function of SMC family genes in sarcoma have not been reported to date. With the development of microarray technology, research methods have also undergone revolutionary changes [10]. Based on the analysis of the expression of thousands of genes, or on copy number variations, recorded in publicly-available databases, we analyzed the expression of different SMC family members in sarcoma and mutations of these genes in this tumor, with the aim of exploring the expression patterns, potential functions and prognostic value of different SMC family members.

## RESULTS

### Transcriptional levels of SMCs in sarcoma

**Figure 1:**
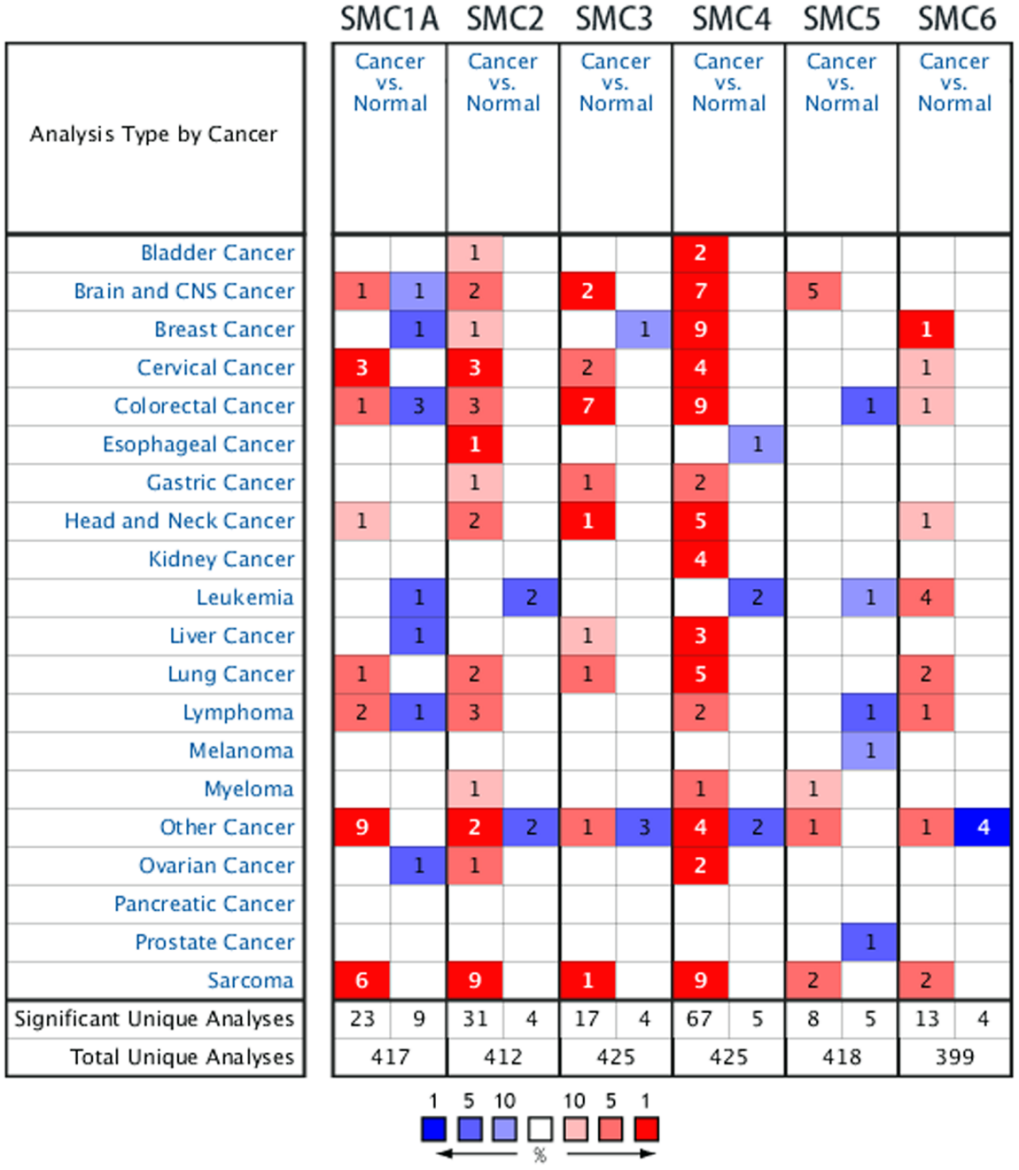
**The Transcription Levels of SMC Factors in Different Types of Cancers (ONCOMINE).**

Previous studies have identified six SMC factors in mammalian cells. In the present study, the ONCOMINE database was used to compare levels of SMC transcripts in cancer and healthy tissue. It was found that SMCs were generally overexpressed in most tumors. All 6 SMC members were particularly highly upregulated in sarcoma tissues (Figure 1). According to these results, SMC1A mRNA was significantly upregulated in sarcoma patients. In the sarcoma dataset of Detwiller et al. [11], the expression of SMC1A in leiomyosarcoma was 4.633-fold higher than in normal issue, and 3.198-fold higher in malignant fibrous histiocytoma. Barretina et al. [12] reported 3.128-, 3.689-, 3.766- and 2.159-fold increased expression of SMC1A in pleomorphic liposarcoma, leiomyosarcoma, myxosarcoma and dedifferentiated liposarcoma, respectively (Table 1).

**Table 1:** The significant changes of smc expression in transcription level between different types of sarcoma (ONCOMINE database).

**Gene ID**	**Types of sarcoma vs normal**	**Fold change**	**p value**	**t test**	**References**
SMC1A	Leiomyosarcoma vs. Normal	4.633	2.21E-7	8.100	Detwiller Sarcoma [11]
	Malignant Fibrous Histiocytoma vs. Normal	3.198	2.86E-6	6.017	Detwiller Sarcoma [11]
	Pleomorphic Liposarcoma vs. Normal	3.128	2.57E-11	10.899	Barretina Sarcoma [12]
	Lieomyosarcoma vs. Normal	3.679	5.95E-12	10.244	Barretina Sarcoma [12]
	Myxofibrosarcoma vs. Normal	3.766	5.84E-13	13.540	Barretina Sarcoma [12]
	Dedifferentiated Liposarcoma vs. Normal	2.159	2.92E-8	8.616	Barretina Sarcoma [12]
SMC2	Myxofibrosarcoma vs.Normal	3.937	1.62E-15	12.726	Barretina Sarcoma [12]
	Pleomorphic Liposarcoma vs. Normal	3.049	2.17E-10	9.161	Barretina Sarcoma [12]
	Myxoid/Round Cell Liposarcoma vs. Normal	2.160	2.15E-9	8.533	Barretina Sarcoma [12]
	Lieomyosarcoma vs. Normal	2.358	3.53E-7	6.172	Barretina Sarcoma [12]
	Fibrosarcoma vs.Normal	6.272	6.17E-6	5.985	Detwiller Sarcoma [11]
	Lieomyosarcoma vs. Normal	6.259	8.84E-6	5.706	Detwiller Sarcoma [11]
	Malignant Fibrous Histiocytoma vs. Normal	8.267	4.09E-6	5.784	Detwiller Sarcoma [11]
	Round Cell Liposarcoma vs. Normal	3.615	7.79E-5	5.149	Detwiller Sarcoma [11]
	Synovial Sarcoma vs. Normal	3.147	3.68E-5	5.192	Detwiller Sarcoma [11]
SMC3	Synovial Sarcoma vs. Normal	5.206	3.75E-9	10.715	Detwiller Sarcoma [11]
SMC4	Lieomyosarcoma vs. Normal	5.750	3.48E-16	14.780	Barretina Sarcoma [12]
	Pleomorphic Liposarcoma vs. Normal	4.898	6.20E-14	12.747	Barretina Sarcoma [12]
	Myxofibrosarcoma vs.Normal	4.866	6.18E-16	13.686	Barretina Sarcoma [12]
	Dedifferentiated Liposarcoma vs. Normal	3.671	8.36E-15	12.550	Barretina Sarcoma [12]
	Myxoid/Round Cell Liposarcoma vs. Normal	2.839	1.52E-7	7.823	Barretina Sarcoma [12]
	Fibrosarcoma vs.Normal	8.252	7.26E-9	9.121	Detwiller Sarcoma [11]
	Lieomyosarcoma vs. Normal	8.448	1.32E-8	9.106	Detwiller Sarcoma [11]
	Pleomorphic Liposarcoma vs. Normal	6.595	4.78E-8	9.134	Detwiller Sarcoma [11]
	Malignant Fibrous Histiocytoma vs. Normal	8.103	5.78E-9	10.267	Detwiller Sarcoma [11]
SMC5	Synovial Sarcoma vs. Normal	3.205	6.02E-5	5.068	Detwiller Sarcoma [11]
	Malignant Fibrous Histiocytoma vs. Normal	3.228	5.58E-5	4.943	Detwiller Sarcoma [11]
SMC6	Malignant Fibrous Histiocytoma vs. Normal	4.214	1.70E-6	6.751	Detwiller Sarcoma [11]
	Lieomyosarcoma vs. Normal	3.745	9.56E-6	5.648	Detwiller Sarcoma [11]

The Barretina datasets [12] also indicated that the expression of SMC2 was increased 3.937-, 3.049-, 2.160- and 2.358-fold in mucinous fibrosarcoma, pleomorphic liposarcoma, circular cell liposarcoma and leiomyosarcoma, respectively. Also in the Detwiller datasets, SMC2 was overexpressed in fibrosarcoma, lieomyosarcoma, malignant fibrous histiocytoma, round cell liposarcoma and synovial sarcoma (6.272-, 6.259-, 8.267-, 3.615-, and 3.147-fold increases, respectively, compared to normal samples, Table 1).

SMC3 was found to be overexpressed in synovial sarcoma (by 5.206-fold in the Detwiller dataset) In the Barretina datasets, SMC4 was overexpressed in lieomyosarcoma, pleomorphic liposarcoma, myxofibrosarcoma, dedifferentiated liposarcoma and myxoid/round cell liposarcoma compared to normal samples (5.750-, 4.898-, 4.866-, 3.671- and 2.839-fold increases, respectively). Also in the Detwiller datasets, SMC4 was overexpressed in fibrosarcoma, lieomyosarcoma, pleomorphic liposarcoma and malignant fibrous histiocytoma compared to normal samples (8.252-, 8.448-, 6.595- and 8.103-fold increases, respectively, Table 1).

In the Detwiller datasets, SMC5 was overexpressed in synovial sarcoma (3.206-fold) and malignant fibrous histiocytoma (3.228-fold) compared to normal samples. Finally, also in Detwiller et al’s datasets, highly expressed SMC6 was found in malignant fibrous histiocytoma (4.215-fold) and lieomyosarcoma sarcoma (3.745-fold) compared to normal samples (Table 1).

### Association between SMC mRNA level and clinicopathological parameters in patients with sarcoma

**Figure 2:**
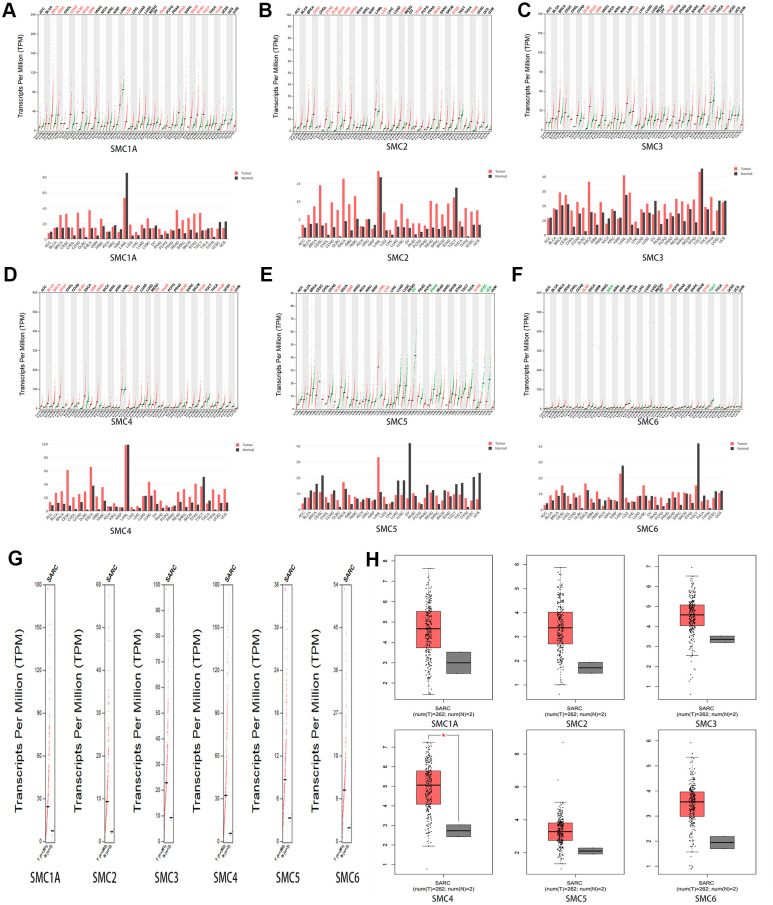
**The Expression of SMCs in Sarcoma (GEPIA).** (**A**)The expression of SMC1A in pan-cancer. (**B**) The expression of SMC2 in pan-cancer. (**C**) The expression of SMC3 in pan-cancer.(**D**) The expression of SMC4 in pan-cancer.(**E**) The expression of SMC5 in pan-cancer.(**F**) The expression of SMC6 in pan-cancer. (**G**, **H**) The expression of SMCs in SARC.

We used GEPIA datasets (http://gepia.cancer-pku.cn/) to compare the expression of SMC mRNAs in sarcoma and normal tissues. It was confirmed that the expression levels of SMC1A, SMC2, SMC3, SMC4, SMC5 and SMC6 in sarcoma were higher than in normal tissues (Figure 2A–2H).

### SMC expression in sarcoma cell lines

**Figure 3:**
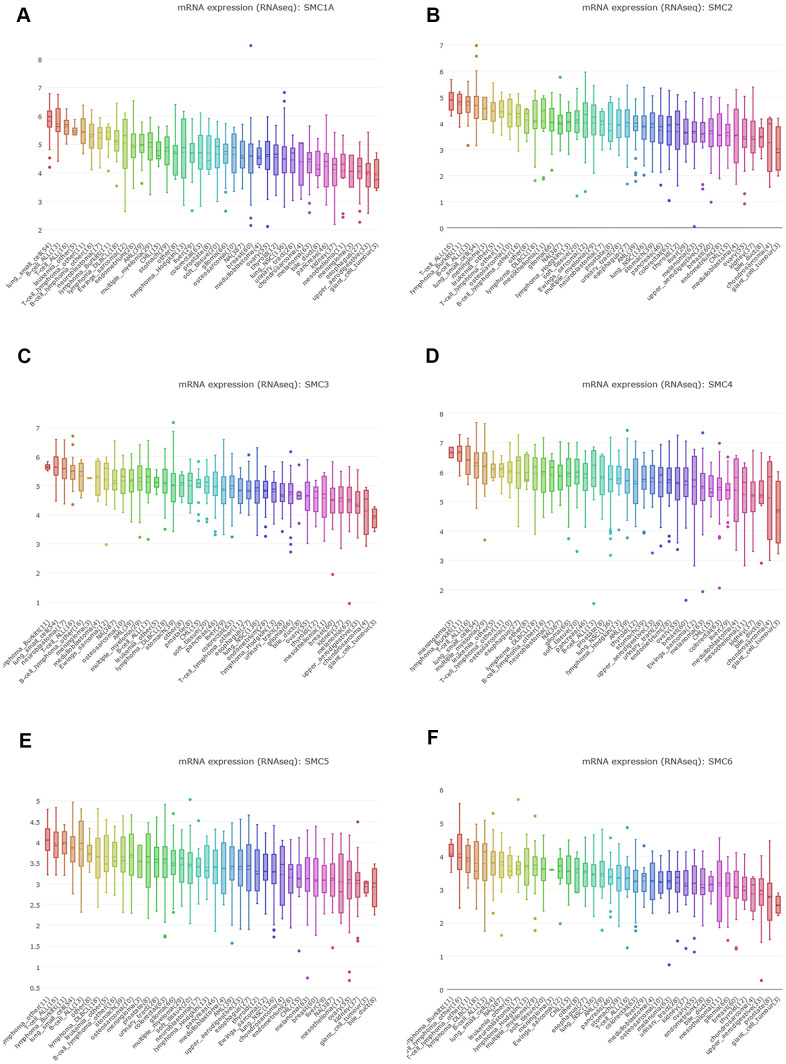
**The Expression of SMCs Sarcoma Cell Lines (CCLE).** (**A**) The expression of SMC1A in sarcoma cell lines, analyzing by CCLE. (**B**) The expression of SMC2 in sarcoma cell lines, analyzed by CCLE. (**C**) The expression of SMC3 in sarcoma cell lines, analyzed by CCLE. (**D**) The expression of SMC4 in sarcoma cell lines, analyzed by CCLE. (**E**) The expression of SMC5 in sarcoma cell lines, analyzed by CCLE. (**F**) The expression of SMC6 in sarcoma cell lines, analyzed by CCLE.

We expanded the detailed and comprehensive annotation process of the preclinical human cancer models by interrogating the CCLE (https://www.broadinstitute.org/ccle). We concluded that SMC1A, SMC2, SMC3, SMC4, SMC5 and SMC6 were all highly expressed in sarcoma cell lines (Figure 3A–3F).

**Figure 4:**
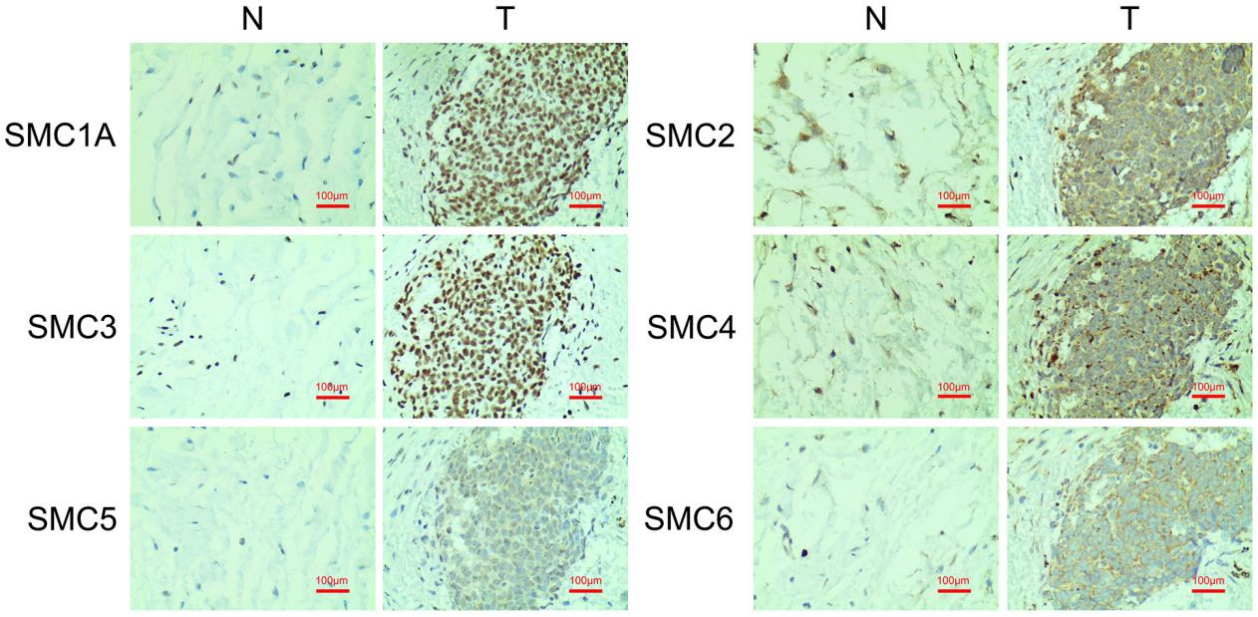
**The Expression of SMCs in synovial sarcoma (Immunohistochemistry).** N: normal control; T: synovial sarcoma.

Additionally, we conducted IHC to confirm SMC expression at the protein level in synovial sarcoma tissues and their counterparts. This revealed that SMC1A, SMC2, SMC3, SMC4, SMC5 and SMC6 proteins were all more highly expressed in the synovial sarcoma tissues than in corresponding normal tissues (Figure 4).

### The prognostic value of SMC expression in sarcoma

**Figure 5:**
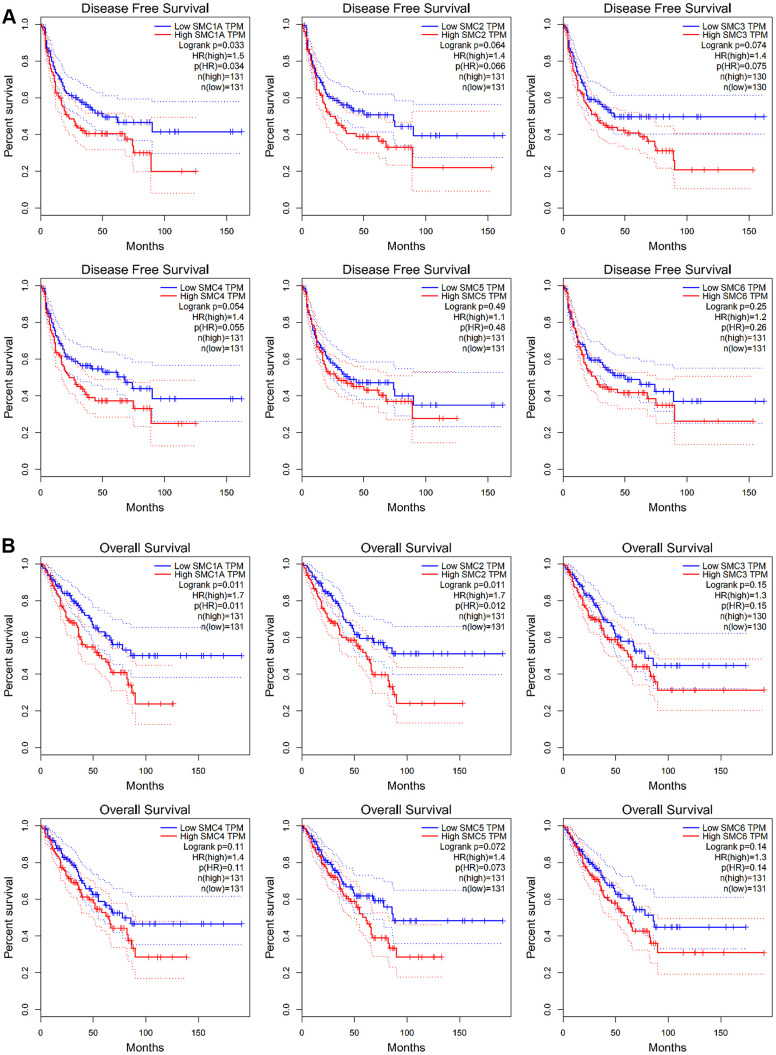
**The Prognostic Value of mRNA Level of SMC Factors in Sarcoma Patients (GEPIA).** (**A**) The prognostic value of mRNA level of SMC factors in disease-free survival (DFS) of sarcoma patients, (**B**) The prognostic value of mRNA level of SMC factors in over survival (OS) of sarcoma patients.

GEPIA was used to investigate the prognostic ability of SMC1, SMC2, SMC3, SMC4, SMC5 and SMC6 expression in sarcoma. In particular, increased expression of SMC1A mRNA were found to be significantly associated with poor DFS (*p*<0.05) and OS (*p*<0.05) in all sarcoma patients (Figure 5A, 5B). Moreover, expression of SMC2 was significantly related to poor DFS (*p*<0.05) (Figure 5B). Increased expression of SMC3, SMC4, SMC5 and SMC6 also tended to be related to poor DSF and OS but without significance (Figure 5A, 5B). Therefore, high expression SMC1A and SMC2 is potentially prognostic for sarcoma.

### Genes co-expressed with SMCs in sarcoma

**Figure 6:**
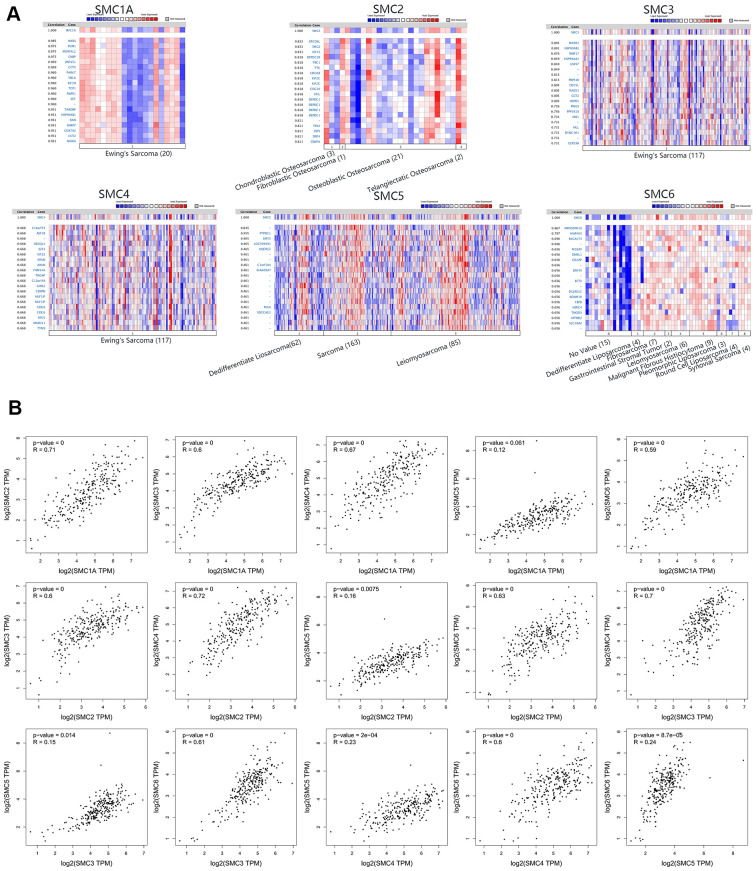
**Co-expressed Genes of SMCs, and the Correction between SMCs in Sarcoma (ONCOMINE and GEPIA). **(**A**) Co-expressed genes of SMCs in sarcoma, analyzed by ONCOMINE. (**B**) The correction between SMCs in sarcoma, analyzed by GEPIA.

We analyzed genes co-expressed with SMC1A in the Ohali dataset. We found that SMC1A was positively connected with NARS, PUM1, MORF4L2, CNBP, ZNF451, CCT5, PARK7, TBCA, EIF3H, TCP1 and RNPS1. For SMC2, we analyzed co-expressed genes in the Kobayashi dataset, and found that its expression was positively corrected with ERCC6L, KIF15, DEPDC18, PRC1, TTK, CDCAB, KIF2C, CDC20, STIL, DEPDC1 and TPX2. For SMC3 in the Postel-Vinay dataset, we found positive connections with MATR3, HSP90AB1, RBM17, HSP90AA1, USP47, PRPF48, CDC5L, RAD21, CCT2, HSPD1 and PRKCI. Genes co-expressed with SMC4 were also analyzed in the Postel-Vinay dataset, revealing that it was positively connected with C16orf75, ASF18, RECQL4, E2F2, KIF22, ANLN, FAM54A, TROAP, C12orf48, GINS2 and CENPN. For SMC5 in the Chibon dataset, positive connections with PTPDC1, SIRT2, LOC399491, UQCRC2, C1orf104, KIAA0907, MGA and SDCCAG3 could be identified. Finally, genes co-expressed with SMC6 in the Detwiller dataset were MPHOSPH10, MAP4K5, B4GALT4, MGEA5, DMXL1, CD2AP, ZNF45, ECT2, DCLRE1C, ADAM10 and CBFB (Figure 6A). Additionally, associations among SMC1A, SMC2, SMC3, SMC4, SMC5 and SMC6 were also analyzed through the GEPIA dataset. Surprisingly, SMC1A was positively connected with SMC2 (R=0.71, *p*<0.05), SMC3 (R=0.6, *p*<0.05), SMC4 (R=0.67, *p*<0.05), and SMC6 (R=0.59, *p*<0.05), but there was no significant correlation between SMC1A and SMC5. SMC2 was positively connected with SMC3 (R=0.6, *p*<0.05), SMC4 (R=0.72, *p*<0.05), SMC5 (P=0.16, *p*<0.05), and SMC6 (R=0.63, *p*<0.05). SMC3 was positively connected with SMC4 (R=0.7, *p*<0.05), SMC5 (R=0.15, *p*<0.05) and SMC6 (R=0.61, *p*<0.05). SMC4 was positively connected with SMC5 (R=0.23, *p*<0.05), and SMC6 (R=0.6, *p*<0.05). Finally, SMC5 was positively connected with SMC6 (R=0.24, *p*<0.05) (Figure 6B).

## DISCUSSION

Bioinformatics is a discipline that exploits the collection, processing, storage, dissemination, analysis and interpretation of biological information [13]. It is also a new subject fusing life science and computer science emerging due to the rapid development of these disciplines [14]. It uses biology, computer science and information technology to reveal the biological mysteries of large and complex biological data.

Bioinformatics analysis has been used to explore the mechanisms of carcinogenesis [15–17]. Previous studies indicated that Uterine Leiomyosarcoma was driven almost exclusively by the inactivation of tumor suppressor genes [18]. Overexpression of SMC factors has been reported in many tumors, and there is evidence for a role of SMC activators in tumorigenesis and prognosis of several cancers [19]. Bioinformatics analysis of sarcoma has not been conducted yet and as far as we aware, the present study is the first to use bioinformatics analysis to investigate the prognostic value and expression of all SMC family members in sarcoma. Moreover, we explored correlations among the six SMCs expressed in human sarcoma. We found that SMC family members likely contributed to carcinogenic effects in the development of sarcoma. Compared with normal tissues, SMC was highly expressed in sarcoma cells. However, only the overexpression of SMC1A and SMC2 was related to the OS of sarcoma patients, whereas SMC3, SMC4, SMC5 and SMC6 had no significant impact on prognosis. Moreover, expression of all SMC family members except SMC1A and SMC5 positively correlated with each other.

The SMC1A gene is located in Xp11.22-p11.21. It consists of 24 introns and 25 exons. Moreover, a key subunit of the cohesin complex is encoded by this gene, which is a significant factor for sister chromatid cohesion [20]. It has been found that these genes are involved in chromosome maintenance and DNA repair. Previous studies reported that upregulated SMC1A might be associated with glioblastoma, lung cancer and colon cancer progression [21–23], but the expression and prognosis of SMC1A in sarcoma had not been investigated to date. In the present study, we found that SMC1A was more highly expressed in sarcoma tissue than in normal tissue in the ONCOMINE and GEPIA datasets, which was confirmed by IHC. As suggested by the CCLE datasets, SMC1A was also highly expressed in human sarcoma cell lines. SMC1A is the core component of sister chromatid cohesion and adhesion complex. SMC1A, SMC3, Mdc1/Rad21, ATPase, and Scc3 subunits form the adhesion protein complex, which play an important role in the correct separation of chromosomes and the repair of DNA double-strand breaks (DSB) [24–27]. Aneuploidy is the main feature of sarcoma. Sarcoma cells usually show abnormal mitotic images, including spindle multipolarity and late chromosomes. During mitosis, the two centrosomes separate and form the two poles of the mitotic spindle. The chromosomes are then captured by the mitotic spindle and finally separated into two daughter cells [28]. If one of the steps is abnormal, it will lead to the occurrence of cell mitosis and multipolarization, leading to severe aneuploidy and sarcoma formation. In summary, SMC1A plays a vital role in the occurrence and progress of sarcoma. In the present study, the prognostic value of SMC1A was confirmed in sarcoma patients. Increased expression of SMC1A was significantly related to poor DFS and OS.

Condensin complex components encoded by the SMC2 gene are essential for the maintenance of chromosomal stability and proper chromosome segregation [29]. SMC2 is involved in mitotic cell division and might have a cancer-promoting effect [30]. SMC2 gene knockdown prevents growth of colorectal cancer [30], and can also increase neuroblastoma cell apoptosis [31]. There are several reports indicating that the expression level of SMC2 mRNA in human pancreatic cancer is significantly higher than in adjacent non-tumor tissues [32, 33]. The SMC2-SMC4 heterodimer as the core, together with three non-SMC proteins, form a condensin complex with 5 subunits. It plays an important role in packaging of chromatin before DNA damage response and cell division, which is essential for maintenance of chromosomal stability and proper chromosome segregation. Previous study indicated that SMC2 might have a pro-oncogenic function and SMC2 was associated with mitotic cell division. Cells harboring SMC2 mutations indicated features with chromosomal destabilization as well, suggesting that impairment of condensin functions by somatic mutation of SMC2 might induce genome instability and contribute to the occurrence and progress of sarcoma [29, 30]. Emerging evidence showed that SMC2 knockdown may suppress growth of sarcoma and increase apoptosis. Several reports indicated that high expression of SMC2 mRNA in human pancreatic cancer tissues than in adjacent non-neoplastic pancreas tissues [34]. In the present study, it was found that the expression of SMC2 in sarcoma was higher than in normal tissues, which was further confirmed using IHC. Additionally, the prognostic value of SMC2 in sarcoma patients was determined by using the GEPIA datasets. Accordingly, increased expression of SMC2 in sarcoma patients was associated with poor OS, in agreement with the role of SMC2 as an oncogene.

The SMC3 gene also encodes an important part of the cohesin complex. Chromosomal instability can be observed in most malignant tumor cells, and the cohesin complex plays a key role in maintaining chromosome stability. Previous studies indicated that loss of the cohesin complex and its cofactors can cause the development of cancer [35–37]. SMC3 is one of the core subunits of the cohesin complex. A previous study indicated that H^2^ inhibited lung cancer progression through down-regulating SMC3. Therefore, we speculated that SMC3 was also associated with the progression of sarcoma. We investigated this in the ONCOMINE and GEPIA datasets, and found that the expression of SMC3 was higher in human sarcoma than in normal tissues. The results of CCLE dataset analysis also showed that SMC3 was highly expressed in human sarcoma cell lines. The prognostic value of SMC3 in sarcoma patients was determined using the GEPIA datasets, revealing that its high expression tended to be associated with poor OS and DFS, but this did not reach statistical significance. SMC4 is located in 3q25.33. Condensin is a heterodimer consisting of SMC2 and SMC4, which is involved in chromatin condensation and gene regulation [38, 39]. There have been several studies finding that the expression of SMC4 was higher in breast cancer, hepatocellular carcinoma, glioma and colorectal carcinoma than in normal tissues [40, 41]. As reported by others, highly expressed SMC4 may upregulate the expression of PLK1, leading to cancer progression and poor prognosis in non-TNBC. In the present study, SMC4 was found to be highly expressed in sarcomas and human sarcoma cell lines. Higher SMC4 expression tended to be associated with poor OS and DFS in sarcoma, but this also failed to achieve significance.

The SMC5/6 complex is the least understood of the SMC complexes, which is essential for eukaryotes. Because it promotes the separation of recombinant intermediates, SMC5-SMC6 plays a prominent role in DNA repair. Recent studies have found that this complex has important functions in the G2 phase of mitosis [42]. There is a view that the loss of the SMC5-SMC6 complex will cause abnormalities in the late stage of replication [43] as it plays a role in the topological structure of DNA [44]. It was reported to play a significant role in repair of damaged DNA [42–45] and removal of toxic structures created by replication [46]. Thus, the role of SMC5-SMC6 in the G2 phase of mitosis may have an impact on cell proliferation, thereby affecting cancer progression, but its function in the occurrence and development of sarcoma remain unknown. In our study, we found that SMC5 and SMC6 were both more highly expressed in sarcoma than in normal tissues, and also in human sarcoma cell lines. Increased expression tended to relate to poor OS and DFS in sarcoma, also without achieving statistical significance.

Bioinformatics analysis is an emerging discipline based on the needs of genomic and proteomic information analysis. It is highly efficient, fast, economical and convenient, and can explore the prognostic value of SMC family genes in a short time. However, some limitations of the present study need to be considered. Although almost all available data were included in our study, the datasets used in this report are nonetheless small, requiring future studies with more datasets to validate the data on a larger sample size in order to better assess the prognostic value of SMC family members in sarcoma.

## CONCLUSIONS

The expression of SMCs in sarcoma was systematically analyzed and prognostic relevance tested. We found that increased expression of SMC1A, SMC2, SMC3, SMC4, SMC5 and SMC6 might play a significant role in sarcoma tumorigenesis. High expression of SMC1A and SMC2 in particular could be useful as molecular markers to identify high-risk patients. Our results also suggest that SMC1A and SMC2 are potential therapeutic targets for sarcoma, while levels of transcripts of SMC3, SMC4, SMC5 and SMC6 are potential prognostic markers to improve the survival rate and prognostic accuracy of sarcoma.

## MATERIALS AND METHODS

### ONCOMINE analysis

The online cancer microarray database ONCOMINE gene expression array datasets (https://www.oncomine.org/) were used to analyze transcription levels of SMCs in different cancers. The Student t-test was used to generate p-values, and the mRNA expression of the SMC family members in clinical cancer samples was compared with normal controls. The cut-off of p-value and fold-change were defined as 0.05 and 2, respectively [47–49].

### GEPIA analysis

GEPIA is a newly developed interactive web server to analyze RNA expression data of 9,736 tumors and 8,587 normal samples from the Cancer Genome Atlas (TCGA) and the Genotype-Tissue Expression (GTEx) projects, using a standard processing pipeline (http://gepia.cancer-pku.cn/). GEPIA can provide customizable functions including tumor or normal differential expression analysis, dimensionality reduction analysis, patient survival analysis, similar gene detection, correlation analysis and profiling according to cancer types or pathological stages [50].

### CCLE datasets

The CCLE (https://www.broadinstitute.org/ccle) project is a cooperation between the Broad Institute and the Novartis Institutes for Biomedical Research and its Genomics Institute of the Novartis Research Foundation. It aims to conduct a detailed genetic and pharmacologic characterization of a large panel of human cancer models, develop integrated computational analyses that link distinct pharmacologic vulnerabilities to genomic patterns and translate cell line integrative genomics into cancer patient stratification [35]. The CCLE provides genomic data, analysis and visualization for approximately 1,000 cell lines. CCLE datasets were used to verify the expression of SMC family members in cancer cell lines [51].

### Immunohistochemistry

Three-μm synovial sarcoma sections were incubated with commercial rabbit polyclonal antibodies against SMC1A-6 (Abcam) at 1/100 dilution overnight at 4° C. Thereafter, the sections were conjugated with horseradish peroxidase (HRP) antibody (1:500 dilution; Santa Cruz Biotechnology, Santa Cruz, CA) at room temperature for 2 h, then covered by DAB (Vector Laboratories, Burlingame, CA), and slides were mounted with Vectashield mounting medium (Vector Laboratories). Subsequently, all fields were observed under light microscopy (Olympus 600 auto-biochemical analyzer, Tokyo, Japan). Control experiments without primary antibody demonstrated that the signals observed were specific.

### Role of the funding source

The study funders/sponsors had no role in the design and conduction of the study, including the collection, management, analysis, and interpretation of the data, preparation, review, or approval of the manuscript, and the decision to submit the manuscript for publication. The datasets used and/or analyzed during the present study are available from the corresponding author on reasonable request.

### Ethics statement

This study was approved by the Second Xiangya Hospital of Central South University Committee for Clinical Research and all methods were in accordance with the Declaration of Helsinki. All the datasets used in the present study were retrieved from the published literature. Therefore, written informed consent was not applicable.

### Availability of data and materials

The datasets used and/or analyzed during the current study are available from the corresponding author on reasonable request.
